# Majority of *Treponema pallidum* ssp. *pallidum* MLST allelic profiles in the Czech Republic (2004–2022) belong to two SS14-like clusters

**DOI:** 10.1038/s41598-024-68656-5

**Published:** 2024-07-29

**Authors:** Eliška Vrbová, Petra Pospíšilová, Eliška Dastychová, Martina Kojanová, Miluše Kreidlová, Filip Rob, Vladimír Vašků, Petra Mosio, Radim Strnadel, Olga Faustmannová, Ivana Kuklová, Monika Dvořáková Heroldová, Hana Zákoucká, David Šmajs

**Affiliations:** 1 Department of Biology, Faculty of Medicine, Masaryk University, Brno, Czech Republic; 2 First Department of Dermatovenereology, St. Annes Hospital and Faculty of Medicine, Masaryk University, Brno, Czech Republic; 3 Department of Dermatovenereology, First Faculty of Medicine and General University Hospital, Charles University, Prague, Czech Republic; 4 Institute of Medical Biochemistry and Laboratory Diagnostics of the General University Hospital and of the First Faculty of Medicine of Charles University, Prague, Czech Republic; 5 Department of Dermatovenerology, Second Faculty of Medicine, Charles University in Prague, Prague, Czech Republic; 6 Department of Medical Microbiology, Faculty of Medicine, St. Anne’s Hospital and Masaryk University, Brno, Czech Republic; 7 National Reference Laboratory for Diagnostics of Syphilis, National Institute for Public Health, Prague, Czech Republic; 8 Department of Dermatovenerology, University Hospital, Brno, Czech Republic

**Keywords:** Microbiology, Clinical microbiology, Microbial genetics, DNA sequencing, Targeted resequencing

## Abstract

Syphilis is a multistage sexually transmitted disease caused by *Treponema pallidum* ssp. *pallidum*. In the Czech Republic, there are around 700–800 new syphilis cases annually, continuously increasing since 2012. This study analyzed a total of 1228 samples from 2004 to 2022. Of the PCR-positive typeable samples (n = 415), 68.7% were fully-typed (FT), and 31.3% were partially-typed. Most of the identified isolates belonged to the SS14-clade and only 6.3% were the Nichols-like cluster. While in the beginning of sample collection isolates have been macrolide-susceptible, recent isolates are completely resistant to macrolides. Among the FT samples, 34 different allelic profiles (APs) were found. Most of the profiles (n = 27) appeared just once in the Czech population, while seven profiles were detected more than twice. The most frequent APs belonged to two separate groups of SS14-like isolates, including group of 1.3.1 (ST 1) and 1.26.1 (ST 25) profiles, and the second group containing 1.1.8 (ST 3), 1.1.1 (ST 2), and 1.1.3 (ST 11) (representing 57.5%, and 25.3% of all detected APs, respectively). Both groups consistently differed in 6 nucleotide positions in five genes (TP0150, TP0324, TP0515, TP0548, and TP0691) coding amino-acid replacements suggesting that one or more of these differences could be involved in the higher success of the first group.

## Introduction

Syphilis is a multistage sexually transmitted disease caused by *Treponema pallidum* ssp. *pallidum* (TPA). In the recent years, syphilis has posed an increasing threat with over 7.1 million new cases a year worldwide^1^. Vulnerable populations include men who have sex with men (MSM), sex workers and their clients, transgender people, young adults, migrant populations, and people living in areas with civil conflicts^2^.

In the Czech Republic, there are around 700–800 new syphilis cases annually, with number of total cases that has increased continuously since 2012^3^. The increasing number of syphilis cases requires increased disease monitoring, especially in pregnant women, where there is a risk of disease transmission to newborns. Molecular typing of TPA provides a better understanding of pathogen epidemiology and pathogen genetic diversity.

The recently introduced multi-locus sequence typing system (MLST,^4^) determines 3 genomic loci (TP0136, TP0548, and TP0705) containing about 30% of the whole-genome sequence diversity^4^ and allows for discrimination between SS14- and Nichols-like groups of isolates^5^. In addition to typing loci, two 23S rDNA loci in individual isolates can be analyzed to detect the presence of one of two mutations leading to macrolide resistance^6,7^. The 23S rDNA locus was historically part of sequencing-based molecular typing (SBMT)^8^. MLST has already been used on TPA isolates from the Czech population^9^ as well as from many other populations, including, China and Japan^10,11^, Cuba^12,13^, Canada^11,14^, Argentina^15^, Madagascar^10,11^, Australia^16^, Switzerland^4^, Spain^17^, France^4,18^, the Netherlands^19^, and others.

As a result of increased TPA molecular typing efforts, an increasing amount of patient data and data regarding allelic variants of individual TPA loci has been recently added to the PubMLST database^11^, allowing assignment and numbering of novel alleles and long-term storage of patient data. As of September 2023, the PubMLST database contained data on more than 1600 TPA isolates and 4700 allele sequences, with 117 different allelic profiles (APs) with assigned allelic numbers.

In this study, we (1) expanded our previous research focusing on MLST typing of clinical samples from patients diagnosed or suspected of having syphilis between 2018 and 2021 and (2) retrospectively re-analyzed previously collected samples to obtain more fully typed MLST profiles from previous decades going back to 2004. Analysis of TPA samples between 2004 and 2022 revealed high levels of TPA isolate diversity in the Czech population, with certain APs being more frequent than others.

## Results

### Clinical samples isolated between the years 2004–2022 from patients diagnosed or suspected of having syphilis.

**Figure 1:**
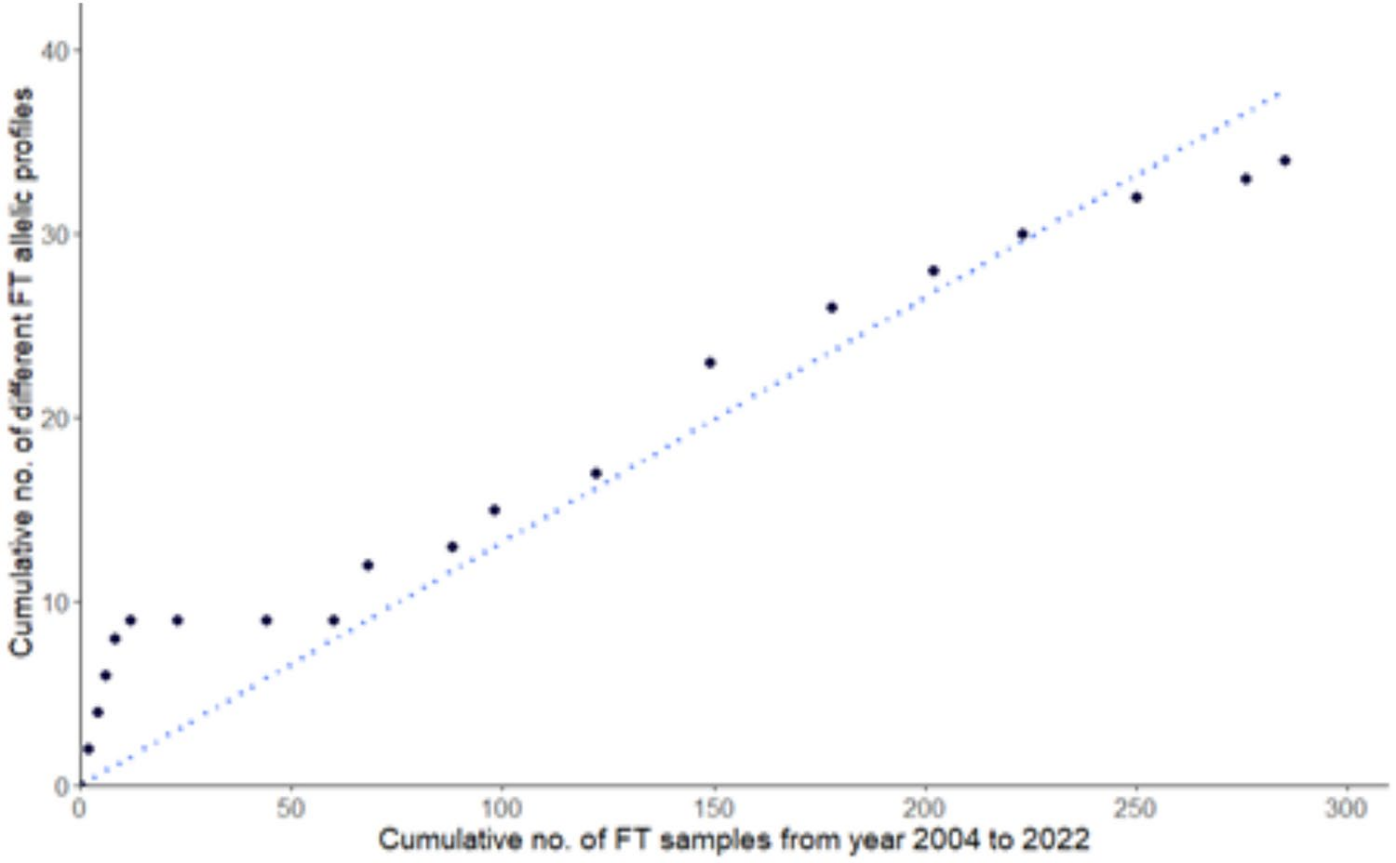
Correlation between the number of examined samples and the number of fully typed allelic profiles.

In total, 1,228 samples were collected from 1054 patients diagnosed or suspected of having syphilis. Out of 1,054 patients, 39.4% contained samples with typeable TPA DNA (n = 415) (i.e., the treponemal sequence was determined at least one MLST locus). From typeable samples (n = 415), 68.7% (n = 285) were fully typable (FT), and 31.3% were partially typable (n = 130). Among the FT samples, a total of 34 different allelic profiles (APs) were found, 13 of them were newly described APs containing new alleles (ST 54—1.30.1, ST 125—1.30.10, ST 66—1.37.1, ST 51—1.38.1, ST 55—1.41.1, ST 52—1.39.1, ST 53—1.40.1, ST 129—1.42.1, ST 130—1.68.1) or new combination of already described alleles (ST 126—1.35.1, ST 127—1.27.1, ST 128—19.26.1, ST 79—6.26.1). In total, 8 new alleles of TP0548 were found (Table S2). The cumulative number of discovered profiles increased with the cumulative number of examined samples (correlation coefficient R^2^ = 0.97, *p* < 0.0001) (Fig. 1).

### Occurrence of macrolide resistance-causing mutations

**Figure 2:**
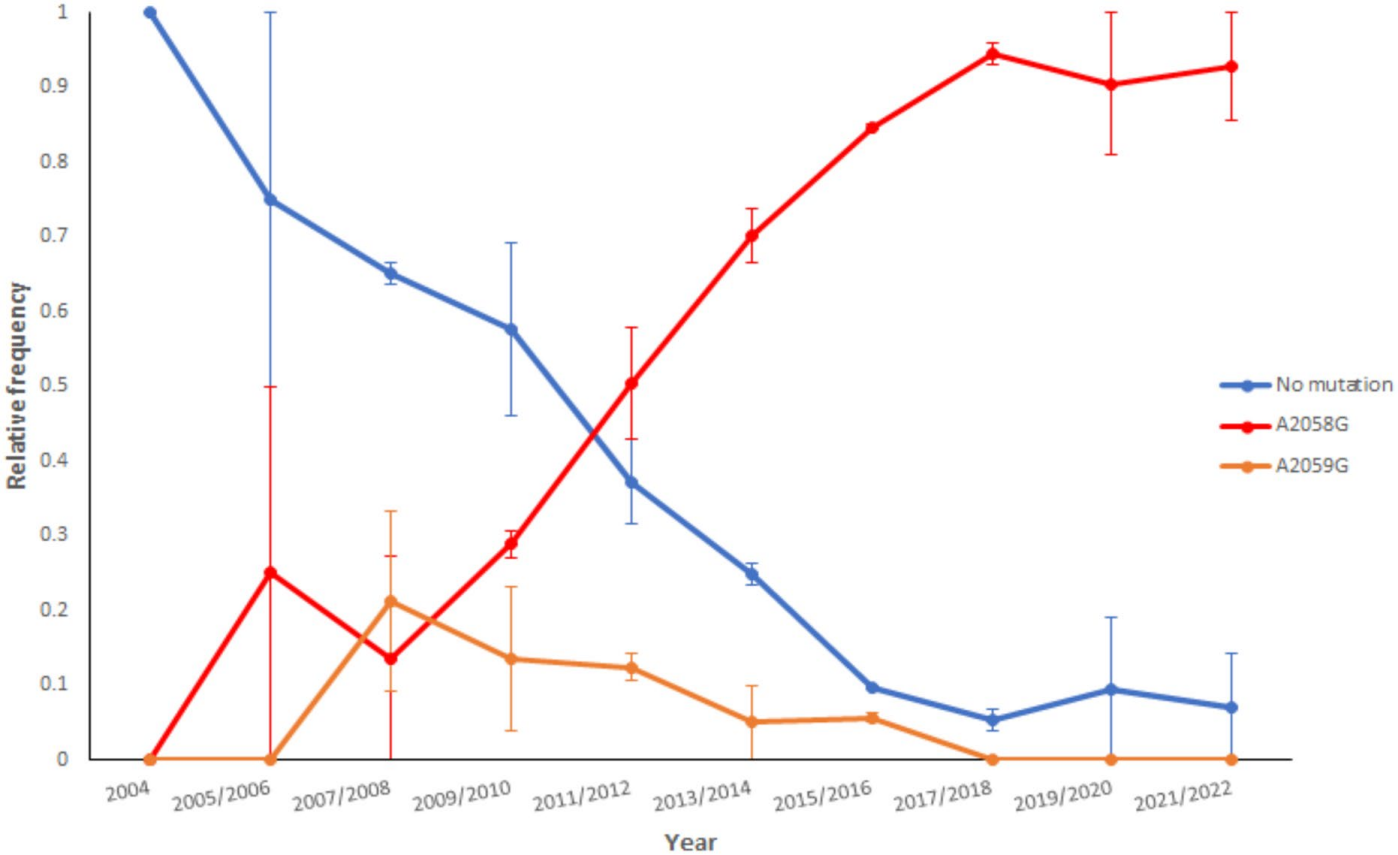
Frequency of mutations causing macrolide resistance in TPA isolates from the Czech Republic. The prevalence over the two-year intervals was calculated as an average; standard errors of the mean are shown. Due to the low number of samples available in 2004, standard errors were not calculated.

During the years 2004–2022, the prevalence of mutations leading to macrolide resistance in 23S rDNA (A2058G or A2059G) changed fundamentally. Over the analyzed years, decreasing susceptibility and increasing resistance were observed. Recently, TPA susceptibility to macrolides in the Czech population is close to zero, while the A2058G mutation is ubiquitous. At the same time, the second macrolide resistance-causing mutation (A2059G) has recently disappeared from the Czech population (Fig. 2).

### Analysis of individual allelic profiles (APs) with respect to patient/isolate characteristics

**Figure 3:**
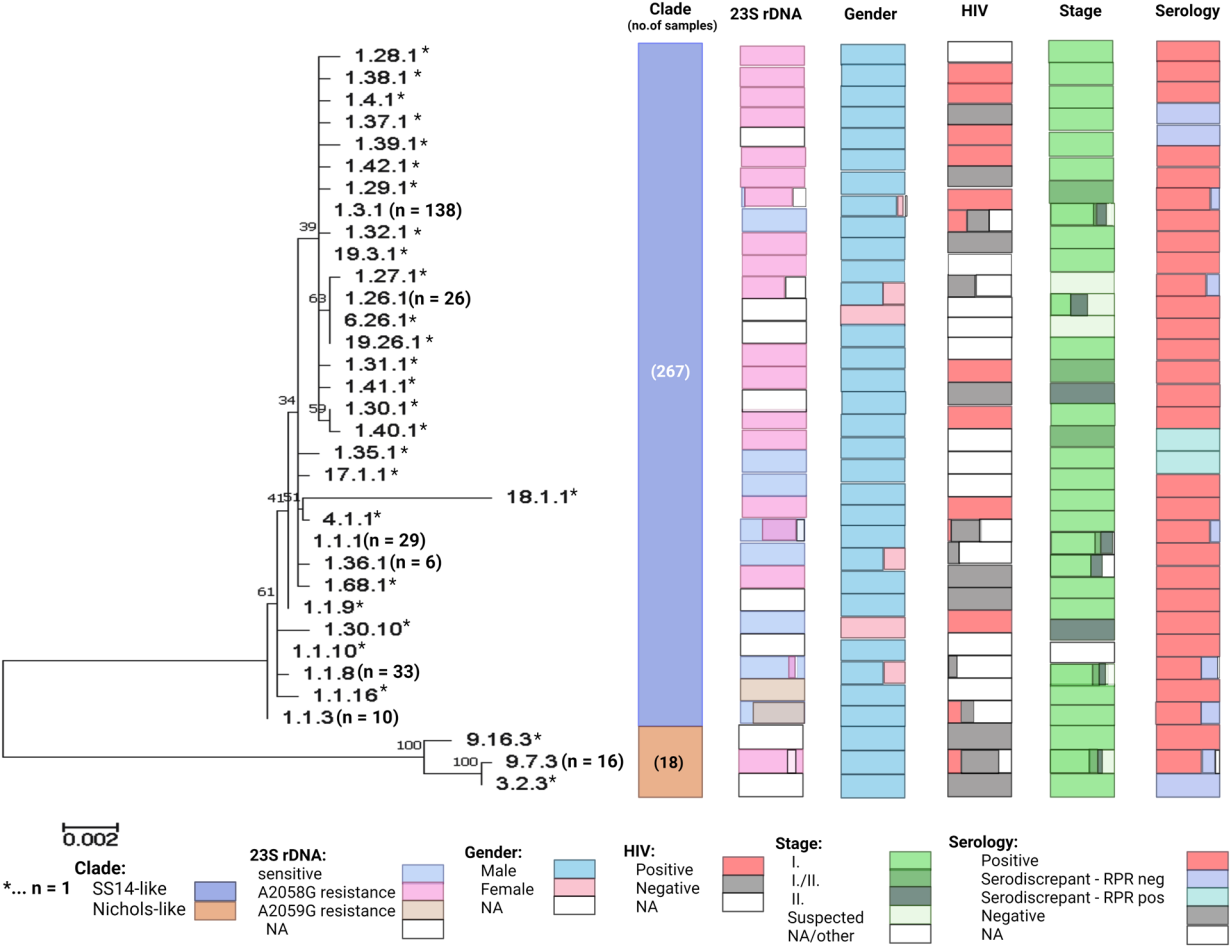
Phylogenetic tree and selected clinical characteristic of detected APs. The evolutionary history was inferred by using the Maximum Likelihood method based on the Tamura-Nei model^30^. The tree with the highest log likelihood (− 4394.49) is shown. The percentage of trees in which the taxa clustered together is shown next to the branches. The tree is drawn to scale, with branch lengths measured in the number of substitutions per site. The analysis involved 34 nucleotide sequences. There was a total of 2585 nucleotide positions in the final dataset. Evolutionary analyses were conducted in MEGA7^31^. AP 18.1.1. contained rearrangements in the TP0136 locus, explaining the special position in the phylogenetic tree was described previously^9^.

Phylogenetic relationships between individual APs are shown in Fig. 3, together with additional patient/isolate characteristics. The detailed characteristics of detected APs are shown in Table S3. All APs detected more than twice in the Czech population were analyzed for possible association with clinical characteristics, including gender, age, city of origin, clinical material, stage of disease, HIV status, serology, individual serological tests, and the presence of mutations causing macrolide resistance. Significant differences in the association (statistical significance was set to *p* = 0.00625 due to multiple comparisons, see Material and Methods) with disease stages were found for AP 1.26.1(ST 25), which was associated with primary syphilis (*p* < 0.0001). Macrolide resistance mutations were associated with profiles 1.3.1 (ST 1) (*p* < 0.0001), 1.26.1 (ST 25) (*p* = 0.005), and 1.1.3 (ST 11) (*p* < 0.0001), respectively, while the absence of mutations encoding macrolide resistance was associated with AP 1.1.8 (ST 3) (*p* < 0.0001), and 1.36.1 (ST 44) (*p* < 0.0001). HIV status was more often found negative for 1.26.1 (ST 25) (*p* = 0.004) compared to other APs.

### Time analysis of prevalence of APs and AP success rates

**Figure 4:**
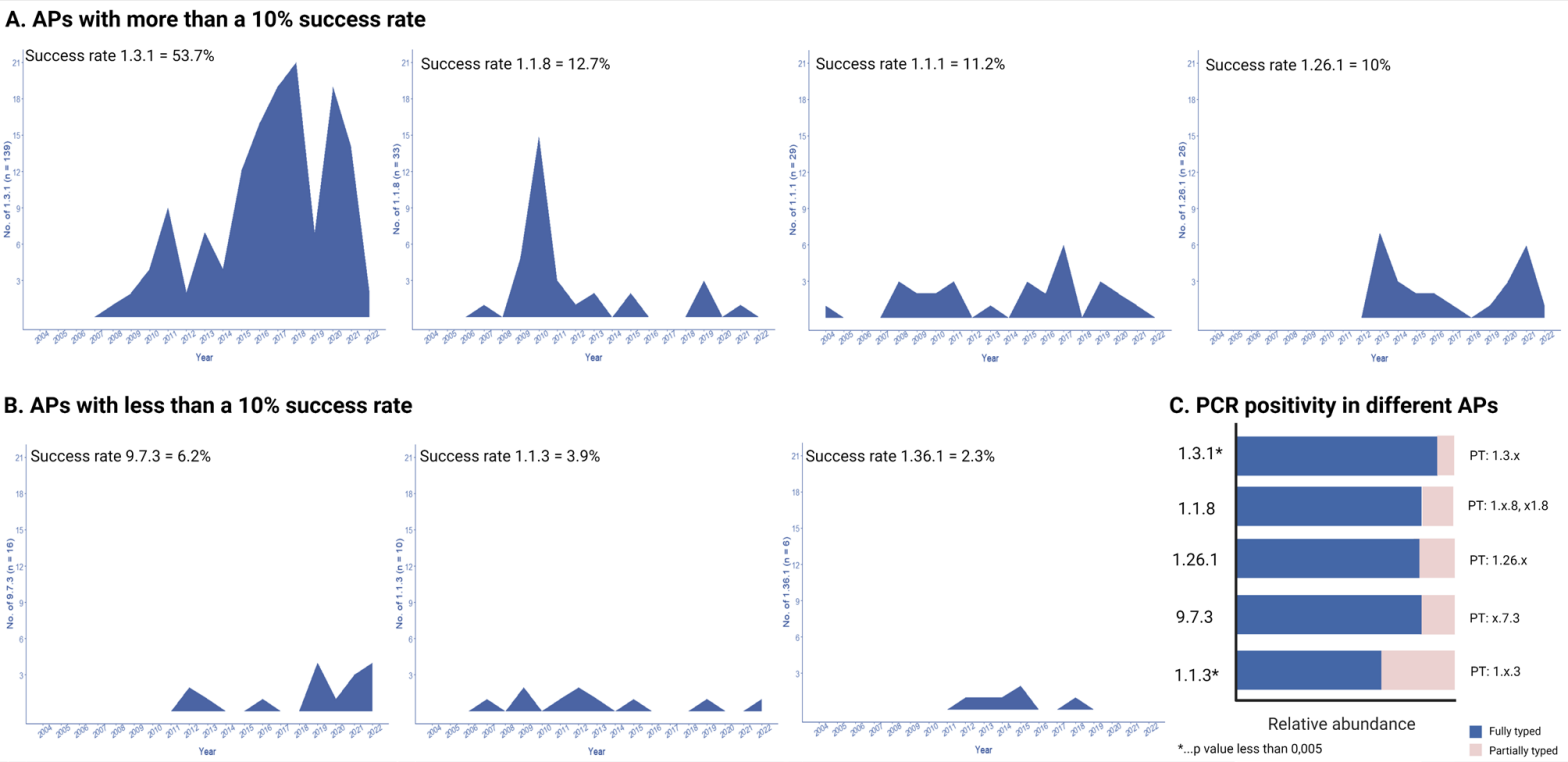
Time distribution of APs, AP success rates, and PCR positivity. (**A**) APs with more than a 10% success rate. (**B**) APs with less than a 10% success rate. The percentage of AP success was calculated from the number of APs that occurred more than twice during the whole study period (n = 258). (**C**) PCR positivity in different APs. PCR positivity is visualized as the ratio between fully typable and partially typable profiles relative to fully typable profiles. Partially typable profiles were associated with fully typable profiles when two alleles (out of three) matched the FT profile and when these two alleles were unique for the FT profile. For this reason, we had to exclude profiles 1.1.1 (ST 2) and 1.36.1 (ST 44) from the analysis.

From 34 detected APs, most of the profiles (n = 27; i.e., ST 6—3.2.3, ST 7—1.4.1, ST 19—1.1.10, ST 27—1.1.9, ST 41—1.29.1, ST 42—1.1.16, ST 43—1.28.1, ST 45—4.1.1, ST 46—1.31.1, ST 48—17.1.1, ST 49—18.1.1, ST 50—1.32.1, ST 51—1.38.1, ST 52—1.39.1, ST 53—1.40.1, ST 54—1.30.1, ST 55—1.41.1, ST 56—19.3.1, ST 66—1.37.1, ST 79—6.26.1, ST 109—9.16.3, ST 125—1.30.10, ST 126—1.35.1, ST 127—1.27.1, ST 128—19.26.1, ST 129—1.42.1, and ST 130—1.68.1) were detected in the Czech population just once, while seven other profiles were detected more than twice during 2004–2022. Time distribution of the latter group of APs is shown in Fig. 4A and B. The most prevalent profile, 1.3.1 (ST 1) (n = 138), was first detected in 2007 and became the most frequent in recent years. The second most frequent profile, 1.1.8 (ST 3) (n = 33), peaked in 2010 and recently has become quite rare in the population, while profiles 1.1.1 (ST 2) (n = 29) and 1.1.3 (ST 11) (n = 10) were only occasionally detected in the Czech population. Of the three newly detected APs in 2012–2013 (i.e., 1.26.1 (ST 25), 1.36.1 (ST 44), and 9.7.3 (ST 26)), AP 1.26.1 (ST 25) (n = 26) has been regularly detected in the population since that time; the prevalence of AP 9.7.3 (ST 26) (n = 16) has been increasing in the recent years, and AP 1.36.1 (ST 44) (n = 6) appears to be slowly disappearing from the population. The percentage of AP success was calculated from the number of APs that occurred more than twice during the full study period (n = 258) and represents the number of individual APs out of all APs (Fig. 4A and B). To evaluate PCR positivity within APs repeatedly observed in the Czech population we compared the number of FT samples and PT samples belonging to the same AP (Fig. 4C). The most prevalent AP, 1.3.1 (ST 1), was significantly more frequently fully typed, while AP 1.1.3 (ST 11) was fully typed with a lower frequency (*p* < 0.005).

## Discussion

In this study, we analyzed samples from syphilis patients in the Czech Republic collected between years 2004 and 2022, which covered 19 years. Compared to a previous study from the Czech Republic covering years 2004–2017^9^, we analyzed almost twice as many clinical samples. The reason for this was (1) the recent increase in the number of syphilis cases in the Czech Republic and an increased number of collected samples and (2) a retrospective re-analysis of older samples using the MLST protocol. Compared to the previous study that reported 48.4% fully typable samples^9^, the improved detection protocol (e.g., using 10 µl of DNA template, and shortening time between swab collection and extraction) used in this study resulted in 68.7% fully typable samples, suggesting that the limited amount of TPA DNA in clinical isolates is a major limitation to TPA typing and that improvements in TPA DNA detection could help to increase the percentage of typeable samples.

Monitoring of the A2058G and A2059G mutations in TPA isolates in the Czech Republic over the past two decades has revealed a dramatic change in the occurrence of macrolide-resistant strains; while almost all strains from the year 2000 were macrolide-susceptible, recent isolates are almost completely resistant to macrolides. A similar trend was found in other countries as well (for review, see Šmajs et al.^20^). Moreover, the A2058G mutation is extremely prevalent, while the A2059G mutation has recently disappeared. The A2058G and A2059G mutations have the same probability of de novo occurrence^20^ and encode resistance to the most commonly used macrolide antibiotics; the A2058G mutation, in contrast to A2059G, does not encode resistance to spiramycin^6,21–25^. Spiramycin prescriptions have consistently decreased over the past two decades in the Czech Republic as described previously^21^, which is likely a factor contributing to the decrease in the occurrence of the A2059G mutation. The other factor adding to the A2059 mutation decrease is its possibly higher fitness cost compared to the A2058G mutation. While differences in the fitness costs of the A2058C and A2059C mutations in the 23S rRNA gene of TPA have not been experimentally demonstrated; however, this phenomenon has been observed in other bacteria^26^. The fact that the A2058G mutation is far more frequent among TPA isolates^27^ supports this explanation. At the same time, increase of prescription of azithromycin could also support the high incidence of A2058G^21^. Moreover, macrolide resistance mutations were found to be positively associated with certain allelic (ST 1—1.3.1, ST 25—1.26.1, and ST 11—1.1.3) and negatively with others (ST 3—1.1.8, and ST 44—1.36.1), likely reflecting the independent emergence of macrolide resistance mutations in different APs^20,28^.

Altogether, 34 different FT APs were identified in this study; most of the APs (78.8%) were detected only once (n = 27). Moreover, the cumulative number of discovered allelic profiles increased with the cumulative number of examined samples (Fig. 1), suggesting that there is a linear correlation between detected TPA genetic diversity (defined as the sequence difference in the detected APs) and the number of tested samples. It can, therefore be expected that further typing of TPA clinical samples will result in additional, new TPA APs, which likely arise from accumulated mutations in the existing profiles. Most of the determined profiles belonged to the SS14-like cluster (Fig. 4), a situation that has been described previously in the Czech Republic and also in other countries^4,9,13,18,19^. At the same time and for unknown reasons, the Czech Republic with only 6.3% of clinical isolates belonging to the Nichols-like cluster^5^ is also a country with one of the lowest prevalence of Nichols-like strains^20,28^.

Out of 34 APs described in this study, 13 have been identified in other countries (Table S4), while 21 were found uniquely in the Czech population (i.e., ST 25—1.26.1, ST 44—1.36.1, ST 41—1.29.1, ST 42—1.1.16, ST 45—4.1.1, ST 46—1.31.1, ST 48—17.1.1, ST 49—18.1.1, ST 51—1.38.1, ST 52—1.39.1, ST 53—1.40.1, ST 54—1.30.1, ST 55—1.41.1, ST 66—1.37.1, ST 79—6.26.1, ST 125—1.30.10, ST 126—1.35.1, ST 127—1.27.1, ST 128—19.26.1, ST 129—1.42.1, and ST 130—1.68.1), likely reflecting a relatively low number of typed samples available from other countries rather than exceptionally high genetic diversity specific to the Czech Republic.

Overall, our study analyzed several times more patient samples compared to other similar studies, with the exception of a recent study from Australia where almost 400 clinical isolates were fully typed and characterized with respect to APs^16^. Moreover, the 19-year time span of our study could have contributed to this finding as well, since the majority of new APs were derived from the most common APs, e.g., in the case of AP 1.3.1, (ST 1), 14 of the 21 unique APs (66,7%) share the AP scheme 1.x.1 (where x > 1), and where additional genetic diversity is predominantly accumulated at the TP0548 locus, which is known to be highly variable but genetically relatively stable^9^. The detection of many APs over the years could thus reflect ongoing genetic diversification of TPA. This also appears to be the case for the fourth most prevalent profile, 1.26.1, in the Czech Republic; 1.26. 1 (ST 25), in the Czech Republic; 1.26.1 (ST25) still appears to be unique to the Czech population despite its first identification in 2013 in Brno and its recent spread to patients whose samples were collected in Prague. AP 1.26.1 (ST 25) differs from AP 1.3.1 (ST 1) in just a single nucleotide position at the TP0548 locus; thus 1.26.1 (ST 25) simple represents a new “version” of AP 1.3.1 (ST 1), which may explain its high success rate (Fig. 4). However, it is not clear if the revealed association of AP 1.26.1 (ST 25) with primary syphilis and negative HIV status reflects genetic or epidemiological differences from AP 1.3.1 (ST 1).

The most prevalent profile in the Czech Republic, 1.3.1 (ST 1), was significantly more frequently fully typed (Fig. 4) compared to 1.1.3 (ST 11), suggesting that the treponemes with successful APs could be present in higher amounts in biological samples and therefore less frequently partially typed. Moreover, if this assumption is correct, the higher number of treponemes present could explain the observed higher success rate of the most prevalent APs including 1.3.1 (ST 1).

Compared to results of other typing studies, the 1.3.1 (ST 1) profile is the most frequent or one of the most frequent among studies from Europe, North and South America, and Australia; however, it appears to be less frequent in Japan (9.6%), although the number of participants in the corresponding study was rather low (n = 52). A study from China analyzing samples from 74 participants did not find the 1.3.1 (ST 1) profile at all. The second most successful group of APs contains 1.1.8 (ST 3) and several related genotypes (1.1.1 (ST 2), 1.1.3 (ST 11), 1.1.9 (ST 27)). In contrast to 1.3.1 (ST 1), AP 1.1.8 (ST 3) has an occurrence of up to 11.6% in Europe, America, and Australia, and, at the same time, is very frequent in China (77%) and Japan (76.9%); this opens the question whether different APs can be dominant in Asia compared to other continents with typed strains and if this distribution reflects differences in epidemiological networks or other population aspects such as occurrence and frequency of human polymorphisms.

From an analysis of the most frequent genotypes in the Czech Republic there are two most frequent SS14-like groups of APs, one including AP 1.3.1 (ST 1) and 1.26.1 (ST 25) and together representing 57.5% of all detected APs and a second group containing AP 1.1.8 (ST 3), 1.1.1 (ST 2), and 1.1.3 (ST 11) collectively representing 25.3% of all APs in the Czech Republic. As revealed by phylogenetic analysis of APs and whole genome sequences, these groups are genetically distinct and belong to different subclusters^14^. A detailed inspection of available whole genome sequences of clinical isolates with the same APs revealed 6 nucleotide positions in five genes (TP0150, TP0324, TP0515, TP0548, and TP0691) that code for amino acid replacements in the corresponding proteins (Table 1). However, since only partial genome sequences were available for several APs (Table 1), additional differences could still exist between SS14-like subclusters. It is likely that one or more of these differences could be involved in the higher success of the first group (containing 1.3.1 (ST 1) and 1.26.1 (ST 25) profiles), suggesting that TPA with different allelic profiles could have different levels of fitness with respect to the frequency of syphilis transmission or efficiency of the infection process. However, more data will be needed to test this hypothesis including data on the whole genome sequences of less successful APs that are, at the same time, highly related to the genomes of 1.3.1 (ST 1) and 1.26.1 (ST 25) isolates.

**Table 1:** Two groups of frequent SS14-like APs (ST 1—1.3.1 and ST 25—1.26.1; ST 3—1.1.8, ST 2—1.1.1, and ST 11—1.1.3) in the Czech Republic and whole genome differences between the groups.

Allelic profiles and selected genomes			1.3.1 (ST1)	1.26.1 (ST 25)	1.1.8 (ST 3)	1.1.1 (ST 2)	1.1.3 (ST 11)
			CW30 (CP034921), CW88 (CP034912)	UZ1974 (CP028438), CW45 (PubMLST)^a^	CW33 (PubMLST)^a^, Japan352xe (CP073502)^a^	PT_SIF0697 (CP016045)^a^, SMUTp08 (CP051888)^a^	UW228b (CP010564)
Success rate in Czech Republic			57.5%		25.3%		
TP0150 (PTS fructose transporter subunit IIA)	nt*	778	A		G		
	aa*	260	Ile		Val		
TP0324 (putative outer membrane protein)	nt	1618	A		G		
	aa	540	Thr		Ala		
TP0515 (LPS-assembly protein LptD)	nt	1366	T		C		
	aa	456	Cys		Arg		
TP0548 (TbuX/FadL-like, long-chain fatty acid transport protein)	nt	154	A		G		
	aa	52	Arg		Gly		
	nt	158	A		G		
	aa	53	Glu		Gly		
TP0691 (segregation and condensation protein ScpA)	nt	89	G		A		
	aa	30	Arg		Lys		

Positions where all APs belonging to each of the two subclusters of the SS14-like group differ are shown. Differences that are only present in individual APs are not shown.

*Nucleotide/amino acid gene/protein position according to the strain Nichols (CP04010.2,^32^); aa, amino acid; ^a^partially sequenced genome.

This study demonstrated a high degree of genetic diversity in TPA isolates in the Czech Republic and the dynamic character of TPA allelic profiles in the infected population over the course of almost two decades. Moreover, MLST typing of clinical samples from patients diagnosed or suspected of having syphilis suggests the existence of important differences among APs with respect to the dissemination of syphilis within the human population.

## Material and methods

### Clinical material

In this study, we used clinical samples collected and analyzed in our previous study^9^ as well as samples collected during years 2018–2022 from clinical departments in the cities of Prague and Brno, CZ (i.e., from the Department of Dermatovenereology, First Faculty of Medicine and General University Hospital, Charles University, Prague, and the National Reference Laboratory for Diagnostics of Syphilis, National Institute of Public Health; the Department of Dermatovenereology, St. Anne´s Faculty Hospital, and the Department of Dermatovenereology, Faculty Hospital in Bohunice;).

We collected 1228 samples from 1054 patients. When multiple samples from one patient were collected during the same visit (or year), the sample giving the best amplification results was used for analysis. Samples collected from one patient but not within the same year were considered separate patients.

In this study we analyzed PCR positive and typeable samples (n = 415). This set of samples included swabs (n = 369), whole blood (n = 43), and tissue samples (n = 3). Patient data included gender (male = 371, female = 44, NA = 1), age (mean age = 34.75, standard deviation (sd) = 10.63, range = 0–71), primary diagnosis (primary stage = 258, secondary stage = 62, stage I./II. = 20, latent stage = 7, congenital syphilis = 4, and suspected syphilis = 390), HIV status (positive = 83, negative = 125, NA = 207), and results of serological examination. There were 339 seropositive patients, 60 serodiscrepant (RPR negative/treponemal specific test-positive (n = 57), RPR positive/treponemal specific test-negative (n = 3)) patients, 8 seronegative patients, and 8 patients with unknown serological results or not tested. Serological tests were slightly different depending on source hospital and included *T. pallidum* particle agglutination (TPPA) or *T. pallidum* hemagglutination (TPHA) tests; the rapid plasma regain (RPR) test, and enzyme-linked immunosorbent assay (ELISA) or Western blot for IgM and IgG. Serological tests were provided by OMEGA Diagnostics (Reinbek, Germany), TEST-LINE (Brno, Czech Republic), and MARDX (Carlsbad, CA, USA).

### Molecular typing of TPA

Clinical material, usually blood or swabs (swab eluates in PBS prepared immediately after sample collection by samples providers when possible, respectively),, was used for DNA isolation using QIAamp DNA Blood Mini Kits; for tissue material, a DNeasy Blood & Tissue Kits (Qiagen, Hilden, Germany) was used as described previously^21^. Isolated DNA was used as a template for PCR amplification of 3 typing loci (TP0136, TP0548, and TP0705) and one additional 23S rDNA locus, which was present in two identical genomic copies in each isolate. When all of the 3 typing loci were successfully determined, sample was labelled as fully-typed and ST number was assigned. If only one or two loci were successfully amplified, it was not possible to assign ST number and sample was marked as partially-typed. The nested PCR protocol we used was described previously^4,7,9,18,21^. Briefly, the outer step PCR mixture, with a final volume of 25 μl, contained 7.3 μl of water, 2 μl of a 2.5 mM deoxynucleotide triphosphate (dNTP) mixture, 5 μl of 5× PS GXL buffer, 0.095 μl of each primer (100 pmol/μl), 0.5 μl of PrimeSTAR GXL polymerase (Takara Bio Europe, France), and 10 μl of isolated DNA. The outer step was run under the following conditions: 94 °C (1 min); 98 °C (10 s), 68 °C (15 s; − 1.0 °C per cycle), 68 °C (1 min and 45 s) for 8 cycles; 98 °C (10 s), 61 °C (15 s), 68 °C (1 min and 45 s) for 35 cycles; and 68 °C (7 min).

The PCR mixture (final volume 25 μl) used for the inner step contained: 20.5 μl of water, 2.5 μl of ThermoPol Reaction buffer, 0.5 μl of a 10 mM dNTP mixture, 0.25 μl of each primer (100 pmol/μl), 0.1 μl *Taq* polymerase (5000 U/ml; New England BioLabs, Ipswich, MA, USA) and 1 μl of the PCR product from the outer step. It was run under the following conditions: 94 °C (1 min), 94 °C (30 s), 48 °C (30 s), 72 °C (1 min and 15 s) for 40 cycles, and 72 °C (7 min). DNA from the TPA strain Nichols (5 pg/μl) was used as a positive control; distilled water was used as a negative control. The list of all primers used for nested PCR is shown in Table S1^4,7^. PCR products were purified using QIAquick PCR Purification Kits (Qiagen, Hilden, Germany) according to the manufacturer´s instruction, with the elution volume set to 100 µl. Sequences were obtained by Sanger sequencing done at Eurofins Genomics Company (Constance, Germany). Analyses of the sequences were performed using Lasergene software (DNASTAR v.7.1.0; DNASTAR, Madison, WI, USA). Sequences were assigned allele numbers and ST numbers through TPA PubMLST database^11^. Sequences of 23S rRNA genes were evaluated at positions corresponding to positions 2058 and 2059 in the 23S rRNA gene of *Escherichia coli* (accession no. V00331), where the A for G substitution has been shown to cause macrolide resistance^6,7,22^. Alleles encoding resistance were marked as A2058G or A2059G, depending on the site of substitution.

### Comparison of whole genomes and selection of genes

Whole genomes representing the most successful allelic profiles from two different SS14-like subclusters were compared. One of the clusters was represented by AP 1.3.1 (whole genomes of CW30 (CP034921) and CW88 (CP034912)), and AP 1.26.1 (whole genome of UZ1974 (CP028438) and draft genome of CW45 (published in PubMLST; breadth coverage 95.53%)). A second cluster was represented by APs 1.1.8 (CW33 (published in PubMLST), and Japan352xe (CP073503); breadth coverage of 71.45% and 99.95%, respectively), 1.1.1 (draft genomes of PT_SIF0697 (CP016045), and SMUTp08 (CP051888); breadth coverage of 99.99%, and 89.12%, respectively), and 1.1.3 (UW228b (CP010564); whole genome). Nucleotide differences between two different SS14-like subclusters were evaluated in positions where (1) all genomes had available genome sequences, (2) nucleotide positions within subclusters were the same, and (3) nucleotide differences coded for amino acid replacements. Nucleotide substitutions present in individual genomes were not evaluated.

### Statistical methods

Correlations of patients' characteristics with TPA allelic profiles were performed using the Student´s t-test for continuous variables (age, value of RPR titer), two-sided Fisher´s exact test for categorical variables (gender, city, clinical material, serology, HIV status, stage of the disease, etc.), and linear regression. Statistical significance of patient characteristics correlated with TPA allelic profiles was adjusted with the Bonferroni correction (for 8 groups) at *p* < 0.00625; for other analyses of statistical significance, *p*-values were set to < 0.05. For analysis of RPR titer, a logarithmic transformation with logarithm base 2 was applied prior to analysis. Statistical analyses were performed using R Statistical Software (v4.1.2; https://www.R-project.org)^29^.

### Ethics statement

This study was approved by the ethics committee of the Faculty of Medicine, Masaryk University (5G/2017) and it was conducted in compliance with the Declaration of Helsinki. All patients provided written informed consent.

## Supplementary Information

Supplementary Information.

## Data Availability

The detailed datasets used and/or analysed during the current study available from the corresponding author on reasonable request.
