# Supplementary material for: Majority of Treponema pallidum ssp. pallidum MLST allelic profiles in the Czech Republic (2004–2022) belong to two SS14-like clusters
Source: Sci Rep. 2024 Jul 29;14:17463. doi: 10.1038/s41598-024-68656-5 (PMC11286942; doi:10.1038/s41598-024-68656-5)
Supplement: Supplementary file 1 — Supplementary Information. [file 41598_2024_68656_MOESM1_ESM.docx]

Supplementary material

**Supplementary Table S1.** List of primers used.

| Locus |  | F primer  (5’-3‘) | R primer  (5‘-3‘) | Length of PCR product | Used as sequencing primers |
| --- | --- | --- | --- | --- | --- |
| TP0136 | External | AACCCGTTAGCGCCCAACAT | TCCCAGCTCAGCCGAATCTC | 1789 bp | No |
|  | Internal | AGTGTCTTCCTCGTCCGTTC | CACGTGGTGGTGTCAAACTT | 1206 bp | Yes |
| TP0548 | External | TGGGGCACTAAACCGGAAGA | TACGGGCATTTGCGGATAGG | 1567 bp | No |
|  | Internal | GCGGTCCCTATGATATCGTGT | GAGACCACTTCAGCCCTACTG | 1065 bp | Yes |
| TP0705 | External | GGTCTATATGCAGCCCTTCTTC | TGCGGCTTATCCTGATGAATAG | 1181 bp | No |
|  | Internal | GCTTGAGAACGATACCGGATAC | TATTCTGCGGCGTTGGATAG | 803 bp | Yes |
| 23S rDNA^*^ | External | CGAAGGGAAGCAGGTGTAGT | GCGCGAACACCTCTTTTTAC  GAACCGTCCCTGAAAACTCA | 1666 bp  1658 bp | No |
|  | Internal | GTACCGCAAACCGACACAG | AGTCAAACCGCCCACCTAC | 629 bp | Yes |

*both copies of 23S rRNA gene were amplified

**Supplementary Table S2.** New alleles of TP0548

| Coordinates (according to allele 1) | 20 | 29 | 33 | 60 | 345 | 362 | 364 | 372 | 516 | 521 | 657 |
| --- | --- | --- | --- | --- | --- | --- | --- | --- | --- | --- | --- |
| *Allele 1* | A | G | G | A | G | A | T | G | A | C | A |
| *Allele 30* | A | A | A | A | G | A | G | G | A | C | A |
| *Allele 37* | A | A | A | A | G | A | T | G | A | C | G |
| *Allele 38* | A | A | A | G | G | A | T | G | A | C | A |
| *Allele 39* | A | A | A | A | G | A | T | G | G | T | A |
| *Allele 40* | A | A | A | A | G | G | G | G | A | C | A |
| *Allele 41* | A | A | A | A | G | A | T | A | A | C | A |
| *Allele 42* | A | A | A | A | A | A | T | G | A | C | A |
| *Allele 68* | G | G | G | A | G | A | T | G | A | C | A |

**Supplementary Table S3.** Detailed characteristics of APs.

| Characteristics | 1.3.1 (n = 138) | 1.1.8 (n = 33) | 1.1.1 (n = 29) | 1.26.1 (n = 26) | 9.7.3 (n = 16) | 1.1.3 (n = 10) | 1.36.1 (n = 6) | Rest (n = 27) |
| --- | --- | --- | --- | --- | --- | --- | --- | --- |
| **Gender (M/F)**  **p value** | 128/9 (1 NA)  0.015 | 22/11  **0.0003** | 29/0  0.03 | 17/9  **0.001** | 16/0  0.23 | 10/0  0.61 | 4/2  0.14 | 25/2  0.75 |
| **Age: mean (sd)**  **Range**  **p value** | 35.4 (9.6)  16-63  0.09 | 35.3 (14.3)  0-61  0.59 | 32.0 (8.8)  16-56  0.21 | 31.9 (9.3)  18-56  0.26 | 38.4 (11.4)  19-59  0.12 | 31.6 (11.9)  20-57  0.41 | 24.7 (14.3)  0-39  0.02 | 33.3 (11.1)  0-51  0.58 |
| **Material (WB/SW)**  **p value** | 3/133 (2 others)  0.72 | 1/31 (1 other)  1.00 | 1/28  0.59 | 0/26  1.00 | 2/14  0.07 | 0/10  1.00 | 0/6  1.00 | 1/26  0.56 |
| **Stage: primary (p value)**  **I/II (p value)**  **Secondary (p value)**  **Other* (p value)** | 97 (0.32)  5 (0.41)  19 (1.00)  17 (4.0) | 23 (0.85)  3 (0.21)  3 (0.59)  4 (1.00) | 21 (0.68)  2 (0.64)  5 (0.57)  1 (0.09) | 8 (**<0.0001)**  -  7 (0.0648)  11 **(0.0002)** | 10 (0.78)  2 (0.18)  1 (0.71)  3 (0.48) | 10 (0.03)  -  -  - | 4 (1.00)  -  1 (0.59)  1 (1.00) | 19 (0.83)  2 (0.37)  3 (1.00)  3 (1.00) |
| **City (Prague/Brno)**  **p value** | 106/32  0.02 | 20/13  0.23 | 23/6  0.29 | 6/20  **<0.0001** | 14/2  0.16 | 6/4  0.49 | 5/1  0.67 | 20/7  0.83 |
| **Macrolides (S/A2058G/A2059G)**  **p value^†^** | 1/107/0 (30 NA)  **<0.0001 (with A2058G)** | 28/3/0 (1 NA)  **<0.0001 (with sensitivity)** | 11/15/0 (3 NA)  0.01 (with sensitivity) | 0/18/0 (8 NA)  **0.005 (with A2058G)** | 0/14/0 (2 NA)  0.02 (with A2058G) | 2/0/8  **<0.0001 (with A2059G)** | 6/0/0  **<0.0001 (with sensitivity)** | 3/14/1 (8 NA)  0.50 (with A2059G) |
| **HIV status (P/N)**  **p value** | 40/48 (50 NA)  0.007 | 0/4 (29 NA)  0.30 | 4/12 (13)  0.41 | 0/12 (14 NA)  **0.004** | 3/9 (4 NA)  0.54 | 2/2 (6 NA)  0.63 | 0/1 (5 NA)  1.00 | 8/9 (10 NA)  0.59 |
| **Seropositive/Serodiscrepant**  **RPR_N/RPR_P**  **p value** | 116/18 (2 NA)  (18/0)  0.15 | 23/9 (1 NA)  (9/0)  0.08 | 25/4  (4/0)  0.80 | 21/5  (5/0)  1.00 | 12/3 (1 NA)  (3/0)  0.72 | 7/3  (3/0)  0.38 | 6/0  -  0.59 | 22/5  (3/2)  0.78 |
| **TPPA (P/N)**  **p value** | 129/0 (5 NA)  0.25 | 32/0 (1 NA)  1.00 | 29/0  1.00 | 23/2 (1 NA)  0.024 | 14/0 (1 NA)  1.00 | 10/0  1.00 | 6/0  1.00 | 23/1 (3 NA)  0.24 |
| **IgG (P/N)**  **p value** | 129/1 (4 NA)  1.00 | 32/0 (1 NA)  1.00 | 28/0 (1 NA)  1.00 | 25/1 (1 NA)  0.26 | 13/0 (2 NA)  1.00 | 9/1  0.11 | 6/0  1.00 | 25/0 (2 NA)  0.25 |
| **IgM (P/N)**  **p value** | 120/12 (2 NA)  1.00 | 30/1 (2 NA)  0.5 | 28/0 (1 NA)  0.15 | 23/3  0.49 | 15/0  0.38 | 7/2 (1 NA)  0.18 | 5/0 (1 NA)  1.00 | 21/6  0.02 |
| **RPR (P/N)**  **p value**  **mean (sd)**  **p value** | 116/18  0.26 | 23/9  0.07 | 25/4  1.00 | 21/5  0.58 | 12/3  0.72 | 7/3  0.21 | 6/0  0.59 | 24/3  0.59 |
|  | 3.8 (2.3)  0.61 | 4.1 (2.1)  0.48 | 3.2 (2.3)  0.20 | 3.9 (2.1)  0.77 | 3.8 (2.6)  0.99 | 2 (1.8)  0.04 | 5.2 (2.7)  0.13 | 3.7 (2.4)  0.89 |

M, male; F, female; NA, unknown value; WB, whole blood; SW, swab; P, positive; N, negative; sd, standard deviation; p value set at 0.0065 after Bonferroni correction, statistically significant values are in bold; *Latent stage, congenital stage, undermined (suspected syphilis); **^†^**p value of best association is presented.

**Suplementary Table S4.** TPA APs also found in other countries according to the PubMLST database (10) **and other studies**

| APs in Czech | America (%/out of) | Asia | Europe | Oceania |
| --- | --- | --- | --- | --- |
| 1.3.1 (ST 1)  (n = 138; 47.7%) | Cuba (84.9% /73)  Canada (57.1% /29)  Peru (50% /6)  Argentina (26.5% /34)(14)  USA (9.4% /32) | Japan (9.6% /52)  China, (0% /74) | Ireland (81.8% /11)  Italy (70% /10)  France (61.7%/ 128)  Portugal (52% /25)  Spain (49.3% /71)  Netherlands (40.9% /220)  Switzerland (40.5% /42)  UK (33.3% /22) | Australia (45% /393)(15) |
| 1.1.8 (ST 3)  (n = 33; 11.6%) | Canada (3.6% /29)*  Argentina (2.9 /34)*(14) | China (77% /74)  Japan (76.9% /52) | Netherlands (5.9% /220)  Spain (5.6% /71)  France (3.9% /128) | Australia (4.6% /393)(15) |
| 1.1.1 (ST 2)  (n = 29; 10.2%) | USA (34.4% /32)  Peru (33.4% /6)  Argentina (26.5% /34)(14)  Canada (3.6% /29)*  Cuba (1.4% /73)* | China, (4.1% /74)  Japan (3.8% /52) | Portugal (28% /25)  Switzerland (21.4% /42)  Netherlands (19.1% /220)  France (14.1% /128)  Italy (10% /10)*  Spain (9.9% /71)  UK (9.5% /22) | Australia (2.8% /393)(15) |
| 9.7.3 (ST 26)  (n = 16; 5.6%) | Argentina (35.3% /34)(14)  USA (9.4% /32) | Japan (1.9% /52)* | Italy (10% /10)*  Netherlands (7.7%/ 220)  France (4.7% /128)  Spain (1.4% /71)*  Switzerland (just PT)  Ireland (just PT) | Australia (4.6% /393)(15) |
| 1.1.3 (ST 11)  (n = 9; 3.2%) | USA (15.6% /32) | Russia (66.7% /3) | Switzerland (4.8% /42)*  UK (4.8% /22)*  Netherlands (0.9% /220) | Australia (3.6% /393)(15) |
| 3.2.3 (ST 6)  (0.35%)* | Canada (3.6% /29)* | China (6.8% /74)  Japan (5.8% /52) | Italy (10% /10)*  UK (9.5% /22)  Netherlands (9.1% /220)  Spain (5.6% /71)  Switzerland (4.8% /42)*  France (0.8% /128)* | Australia (2.5% /393)(15) |
| 1.1.10 (ST 19)  (0.35%)* | Cuba (1.4% /73)*  USA (3.1% /32)* | - | Netherlands (1.8% /220) | - |
| 1.1.9 (ST 27)  (0.35%)* | USA (3.1% /32)* | - | UK (4.8% /22)*  Netherlands (1.4% /220)  France (0.8% /128)* | Australia (1.3% /393)(15) |
| 19.3.1 (ST 56)  (0.35%)* | Canada (just PT) | - | Ireland (9.1% /11)*  Netherlands (1.8% /220) | - |
| 1.32.1 (ST 50)  (0.35%)* | Argentina (2.2% /34)* | - | - | Australia (0.5% /393)(15) |
| 9.16.3 (ST 109)  (0.35%)* | USA (3.1% /32)* | - | - | Australia (0.5% /393)(15) |
| 1.28.1 (ST 43)  (0.35%)* | Canada (3.6% /29)* | - | - | - |
| 1.4.1 (ST 7)  (0.35%)* | - | - | Switzerland (7.1% /42) | - |

*n = 1
